# Ubiquitin-Specific Protease 43 Impacts Pancreatic Ductal Adenocarcinoma Prognosis by Altering Its Proliferation and Infiltration of Surrounding Immune Cells

**DOI:** 10.1155/2023/4311388

**Published:** 2023-04-02

**Authors:** Ziqi Zhao, Zhikun Lin, Xin Guo, Abdullah Al-danakh, Hui He, Henan Qin, Chi Ma, Ningning Zhang, Guang Tan

**Affiliations:** ^1^Department of General Surgery, The First Affiliated Hospital of Dalian Medical University, Dalian, China; ^2^Liaoning Key Laboratory of Molecular Targeted Drugs in Hepatobiliary and Pancreatic Cancer, Dalian, China; ^3^Department of Urology, The First Affiliated Hospital of Dalian Medical University, Dalian, China; ^4^Department of Oncology, The First Affiliated Hospital of Dalian Medical University, Dalian, China; ^5^Department of Hematology, The First Affiliated Hospital of Dalian Medical University, Dalian, China

## Abstract

**Background:**

Pancreatic ductal adenocarcinoma (PDAC) is a devastating cancer, and the therapy options for PDAC remain restricted. The distinctive tumor immunological microenvironment (TIME) of PDAC, comprising a high number of stromal cells and a limited infiltration of cytotoxic T lymphocytes (CTLs), rendered immunotherapy ineffective. The protein level of ubiquitin-specific protease 43 (USP43) was a prognostic predictor in numerous cancers; however, its function in PDAC is limited. This article focuses on the influence of USP43 expression on PDAC prognosis and TIME alteration.

**Methods:**

Based on TCGA database and tissue microarray staining, the expression of USP43 in PDAC was evaluated. The association between USP43 and prognosis was then investigated using tissue samples and online databases. In PDAC tumor tissues, the correlation between USP43 expression and clinicopathological characteristics, immune cell infiltration, and prognosis was investigated. The expression of USP43 in PDAC cell lines was evaluated using quantitative polymerase chain reaction. Using a cell counting kit-8 (CCK-8) and a cell colony formation test, the viability of the cells was determined. On the basis of online databases and tissue samples, the link between USP43 and immune cell infiltration around PDAC was also examined. For statistical analyses, the software GraphPad, R, and SPSS 26.0 were utilized.

**Results:**

The expression of USP43 was considerably higher in PDAC compared to normal pancreatic tissue in both the TCGA database and the tissue microarrays of PDAC patients (*P* < 0.001). High USP43 expression was associated with poor overall survival in both the TCGA database and the tissue microarray of PDAC patients (*P* = 0.046 and 0.021, respectively). USP43 overexpression promoted PANC-1 cell proliferation (*P* = 0.0018), but USP43 knockdown decreased PANC02 cell proliferation (*P* < 0.001). According to the TCGA database, USP43 is associated with T cell activation and inhibits CD8^+^ T cell activation in PDAC, as proven by a study of cell lines. Moreover, in both TCGA and PDAC cell lines, USP43 expression was negatively linked with the chemokine signaling pathway.

**Conclusions:**

Overexpression of USP43 is a potential prognostic indicator for PDAC patients. USP43 is a potential biomarker associated with T cell activation, suppression of CD8^+^ T cell enrichment, and the cytokine signal pathway. Future multicenter studies are needed to confirm our findings and their potential application in the treatment of PDAC patients.

## 1. Introduction

Pancreatic ductal adenocarcinoma (PDAC) is a fatal illness that accounts for ∼90% of pancreatic adenocarcinoma and has a 5-year survival rate of 10% [1, 2]. PDAC is highly aggressive and biologically diverse, and patients' prognoses and therapy responses are highly varied, hence effective treatment of PDAC remains limited [3]. Nearly 80% of patients with PDAC are identified with locally advanced disease or distant metastases, restricting their surgical options. Currently, FOLFIRINOX (folinic acid, 5-fluorouracil, irinotecan, oxaliplatin) chemotherapy regimens are the mainstay of treatment for advanced PDAC, with resectable PDAC outcomes improving [4]. Immunotherapy has recently made major advances in the treatment of numerous types of malignancies, which have been found to extend the survival of patients with melanoma, nonsmall cell lung cancer, and renal cell carcinoma [5–8]. Therefore, understanding the biology, microenvironment, and immunotherapy of pancreatic cancer is necessary.

PDAC has a distinct tumor immune microenvironment (TIME) with up to 80% nontumor stroma that includes extracellular matrix proteins (collagens, fibronectin, laminin, glycosaminoglycans) and stromal cells (immune cells, cancer-associated fibroblasts (CAFs), endothelial cells) [9–11]. These cells and structures serve as a physical barrier that prevents immune cells from identifying and destroying PDAC. In addition, the immune cells infiltrating PDAC are uncommon and predominantly suppressive immune cells, such as regulatory T cells (Tregs), myeloid-derived suppressor cells, dendritic cells, and tumor-associated macrophages, while cytotoxic T cells appear to have diminished [12–14]. In addition, a complex network between chemokine and chemokine receptors has been shown to impact TIME in PDAC, hence encouraging cancer growth [15]. Thus, PDAC is considered as “immune desert” or immunologically “cold” tumor. Consequently, it is essential to identify intrinsic and/or extrinsic tumor targets that can alter the TIME of PDAC from immunologically “cold” to “hot” and thereby improve its responsiveness to ICIs.

Most proteins undergo acetylation, methylation, phosphorylation, glycosylation, sumoylation, or ubiquitination after synthesis. One of these alterations is ubiquitination, which enables a small molecule of ubiquitin to attach to lysine residues of target proteins; dysregulation of ubiquitination may contribute to the development of malignancies [16, 17]. Ubiquitination is a reversible process that removes ubiquitin chains from proteins through the function of deubiquitinases (DUBs) and regulates the stability or activity of these proteins through this process [18, 19]. Ubiquitin-specific proteases (USPs) constitute the most numerous categories, and a growing number of studies have shown that USPs influence tumor formation, such as cell proliferation and death [17, 20]. Previous research has revealed that ubiquitin-specific protease 43 (USP43) can greatly influence breast cancer growth and metastasis by regulating EGFR/PI3K/AKT and that USP43 can also regulate the breast cancer cell cycle and epithelial–mesenchymal transition pathway, hence promoting tumorigenesis [21, 22]. Furthermore, USP43 was highly expressed in colorectal cancer (CRC) and increased CRC cell proliferation, invasion, and migration [23]. However, research on the expression of USP43 and its relationship to PDAC is scarce.

In this study, we will investigate the expression of USP43 in PDAC and the relationship between USP43 and patient survival, as well as the effect of USP43 expression on TIME and the likely pathways involved in tumorigenesis and progression.

## 2. Materials and Methods

### 2.1. Study Cohort

This study included a total of 38 PDAC patients treated at the First Affiliated Hospital of Dalian Medical University from June 2019 to December 2021. Participants in this trial had R0 pancreaticoduodenectomy, and all tumor tissues were pathologically confirmed to be PDAC without any prior neoadjuvant therapy. The following clinicopathological characteristics were analyzed: age, gender, T and N stages (according to the 8th edition of the TNM staging system), stage (according to the 2022 version of the NCCN guideline), grade, surrounding organ infiltration, and surrounding fat infiltration. Four plasma tumor indicators, CA19-9, CA125, CEA, and AFP, were investigated. Before enrolment, all patients signed written consents based on their knowledge of the study's nature. The median age of the patients was 64 years old, and overall survival (OS) ranged from 6 to 39 months.

### 2.2. Bioinformatic Analysis

The expression levels of USP43 in various types of cancers were analyzed with the online database (https://xenabrowser.net/datapages/, accessed December 16, 2022) including TCGA (The Cancer Genome Atlas) and GTEx (Genotype-Tissue Expression). In total, 183 samples of PAAD tissues from TCGA, and 167 samples of normal pancreatic tissues from the GTEx dataset were included in this study.

### 2.3. Immunohistochemical (IHC) Staining

Thirty-eight PDAC patients' tumor tissues and normal tissues were provided by Dalian Medical University's First Affiliated Hospital in accordance with Dalian's ethics committee for human research (registration number is PJ-KS-KY-2021-203) and the patient's written consent was taken. The newly dissected tissue (about 3 mm thick) was preserved with 4% paraformaldehyde at room temperature overnight. Afterward, the tissue was washed with running tap water for 5 min before being dehydrated with 75% alcohol, 85% alcohol, and 95% alcohol for 5 min each, followed by three times with 100% alcohol for 5 min each. The tissue was cleared in xylene twice for 5 min each time and then immersed in paraffin three times for 5 min each time. The paraffin-embedded tissue was sectioned on a microtome at 5–8 *μ*m thickness and floated in a 40°C water bath containing distilled water. We transferred the sections onto glass slides suited for immunohistochemistry, allowing the slides to dry overnight before storing them at room temperature. IHC staining was performed according to a standard protocol routinely (KIHC-5, ProteinTech, Wuhan, China). USP43 primary antibody (1 : 50, PA5-55684, ThermoFisher, USA) was used in staining. Then, stained samples were analyzed using standard light microscopy.

Each sample was examined twice by two pathologists who were blinded to the experiment. The expression of USP43 was thus evaluated as follows: the intensity of staining was scored as 0 (negative), 1 (weak), 2 (moderate), and 3 (strong), and the proportions of positive cells were categorized as 0 (<5%), 1 (5%–<25%), 2 (25%–<50%), 3 (50%–<75%), and 4 (75%–100%). The multiplication for intensity and proportion was used to evaluate the level of USP43 expression. ImageJ (version 1.52) was used for the analysis and the cutoff value of USP43 was 2.75. A score <2.75 was defined as low expression, while a score >2.75 was defined as high expression.

Using the same method, each section was stained with different immune cell markers as follows: CD4 (1 : 100, EP204, CST, USA) for helper T lymphocytes (Th cells), CD8 (1 : 100, D8A8Y, CST, USA) for cytotoxic T lymphocytes (CTLs), CD68 (1 : 200, D4B9C, CST, USA) for macrophage, CD163 (1 : 300, D6U1J, CST, USA) for M2-polarized macrophages, FOXP3 (1 : 100, D6O8R, CST, USA) for Tregs. We selected six areas with abundant immune cell infiltration at a high-power field (HFP) (400x), and counted positive cells number in each field, and calculated the average count per HFP.

### 2.4. Cell Culture

Human pancreatic cancer cell lines (BxPC-3, PANC-1) and HEK293T cell line were purchased from Cell Bank of Chinese Academy of Sciences (Shanghai, China). Murine pancreatic cancer cell line (PANC02) was purchased from BeNa Culture Collection (Xinyang, Henan, China). Cells were cultured in a humidified incubator with 5% CO_2_ at 37°C in Dulbecco's modified Eagle's medium (DMEM, C11995500BT, Gibco, USA) containing 10% fetal bovine serum (FBS, LV-FBS-S500, Newzerum, New Zealand) and 1% penicillin–streptomycin (C0222, Beyotime, China).

### 2.5. Lentiviral shRNA Infection

Lentiviral particles generated with a standardized protocol were used to produce the highly purified plasmids. Take 100 mm cell-culture dish as an example, HEK293T cells were transfected with 4 *μ*g of lentiviral shRNA or USP43 plasmid (control group transfected equal plasmid), 3 *μ*g of viral packaging plasmid pPAX2, and 1 *μ*g of envelope plasmid pMD2.G to produce lentivirus. Transfection was performed with LipoFiter Liposomal Transfection Reagent (HB-TRLF-1000, Hanbio, China) and Opti-MEM (31985062, Gibco, USA). During the first 24 hr after infection, no FBS was added to DMEM. We then changed the growth medium with DMEM containing 10% FBS and cultured HEK293T cells for another 24 hr. The virus was collected after being filtered via 0.45 *μ*m filters. When the cancer cells (PANC-1, PANC02, and BxPC-3 cells) covered 70%–80% of the dishes, they were infected with the virus in the presence of polybrene (0.5 *μ*g/ml, 40804ES76, Yeasen, China), and 48 hr after infection, the cells were chosen with 0.5 *μ*g/ml puromycin (QLL-42-01, InvivoGen, Hong Kong, China). USP43 overexpression system was constructed in PANC-1 cells using Lentiviral (Fenghbio, China). PANC02 cells were infected with pPLK/GFP + Puro-Usp43 shRNA using the same method (PPL, China).

### 2.6. Quantitative Polymerase Chain Reaction (qPCR)

Total RNA was extracted using RNAiso Plus (9109, Takara, Japan). According to the manufacturer's instructions, cDNA was synthesized from mRNA using Hifair III 1st Strand cDNA Synthesis SuperMix for quantitative polymerase chain reaction (qPCR) (11141ES60, Yeasen, China). All samples mRNA expression were quantified using the Applied Biosystems® 7,500 Fast system (ThermoFisher, USA) to determine the comparative CT and were normalized to *β*-ACTIN/*β*-Actin levels concentrations. Sequences of primers are reported in Table 1.

### 2.7. Cell Colony Formation

For cell colony formation assays, we utilized DMEM with 10% FBS and 1% penicillin–streptomycin, then we resuspended and counted the cells. Mix of 2 ml medium and 500 cells were added to one well of six-well plate. Repeat it three times for each cell line. We added 100 *μ*l of the abovementioned medium to every well and everyday. After 5–10 days, cell colony had formed and fixed with 4% paraformaldehyde (P0099, Beyotime, China) for 20 min and stained using crystal violet (C8470, Solarbio, China) for 5 min. Then, the pictures were taken under a digital camera and the cell colony formation rate was calculated.

### 2.8. CCK-8

After cell lines were digested and centrifuged, they were resuspended, counted using a cell counting plate. Each well in a 96-well plate was filled with 100 *μ*l cell suspension containing 2,500 cells. The following day, 10 *μ*l of CCK-8 solution (C0041, Beyotime, China) was added to the medium of the first two pairs of wells. Then, continue to culture them for 2 hr in the incubator. After the crystals were completely melted, the absorbance at OD 450 nm was measured to calculate cell proliferation. Each group was replicated three times, and each replication was monitored at four-time points.

### 2.9. Statistical Methods

The clinical data were examined and compared using the *χ*^2^ test, and the Kaplan–Meier technique and log-rank test were used to compute and assess the survival analysis. The Cox proportional hazards model was used to conduct univariate and multivariate Cox regression analysis. Student's *t*-test (paired or unpaired) was used to compare two groups; one-way ANOVA was used to compare more than two groups. Bars and error represent mean ± standard deviations (SD) of replicate measurements. Using the web program Cutoff Finder [24], optimal cut-off values for gene expression were calculated, and the cut-off values served as the basis for classifying them into a higher-level group and a lower-level group. GraphPad Prism 8 software, R software (version 4.0.1), Xiantao Scholarship (https://www.xiantao.love/), and SPSS 26.0 statistical tools were utilized for statistical analyses (IBM). *P*-values < 0.001 ( ^*∗∗∗*^), <0.01 ( ^*∗∗*^), or <0.05 ( ^*∗*^) were considered statistically significant.

## 3. Results

### 3.1. USP43 Is Highly Expressed in PAAD and Correlate with Poor Prognosis

**Figure 1:**
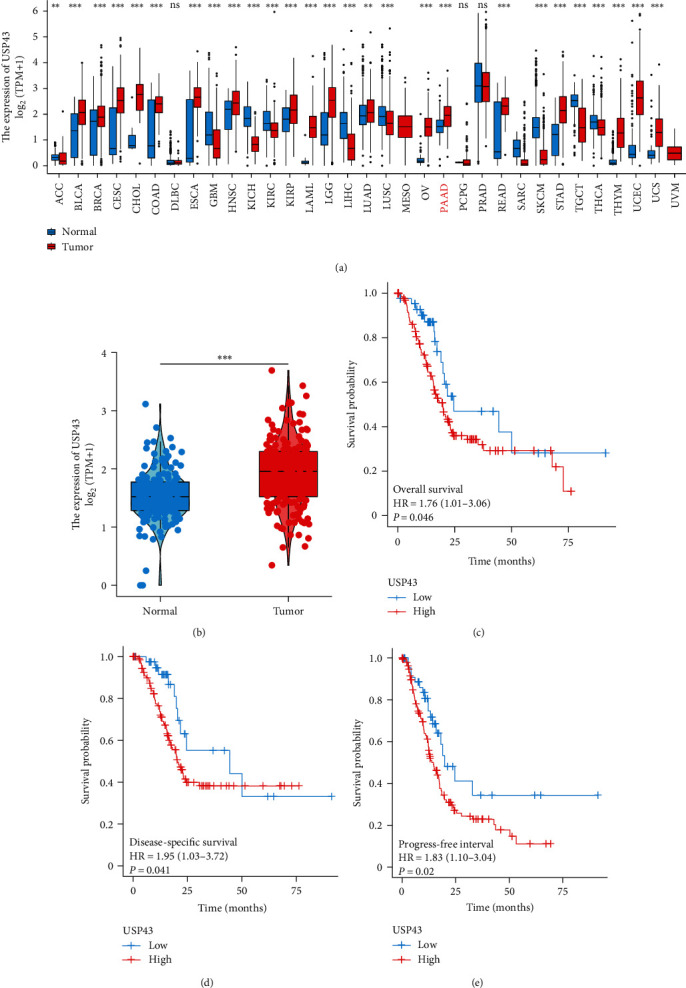
USP43 is highly expressed in PAAD and correlate with poor prognosis based on TCGA database: (a) based on TCGA database, we compared 33 kinds of tumor, and the expression of USP43 was significantly higher in 18 kinds of tumor tissues than normal tissue; (b) based on TCGA database, we analyzed the expression of USP43 in 350 PDAC cases (tumor tissues and normal tissues); (c) according to TCGA databases, we underwent Kaplan–Meier curve to evaluate OS; (d) DSS; and (e) PFI in USP43 high and low group of PDAC patients.  ^*∗∗*^*P*-values < 0.01,  ^*∗∗∗*^*P*-values < 0.001.

Using the TCGA database, the expression of USP43 was studied in 33 types of cancer in order to compare the expression of USP43 between tumor and normal tissues. USP43 expression was higher in tumor tissues than in normal tissues in several types of tumors, including pancreatic adenocarcinoma (PAAD), rectum adenocarcinoma (READ), colon adenocarcinoma (COAD), cholangiocarcinoma (CHOL), stomach adenocarcinoma (STAD), and esophageal carcinoma (ESCA). The difference between PAAD tumor tissue and normal tissues is significant among these malignancies (*P* < 0.001, Figure 1(a) and 1(b)). Then, we collected 350 PDAC patients from the TCGA database and divided them into two groups based on the median expression of USP43 (high vs. low). The relationship between USP43 expression and OS, disease-specific survival (DSS), and progress free interval (PFI) was then investigated, revealing that high USP43 expression was associated with poor OS, DSS, and PFI (*P* = 0.046, HR = 1.76, Figure 1(c)), (*P* = 0.041, HR = 1.95, Figure 1(d)), and (*P* = 0.02, HR = 1.83, Figure 1(e)), respectively.

### 3.2. Validation of USP43 Expression in PDAC Based on Tissue Samples

**Figure 2:**
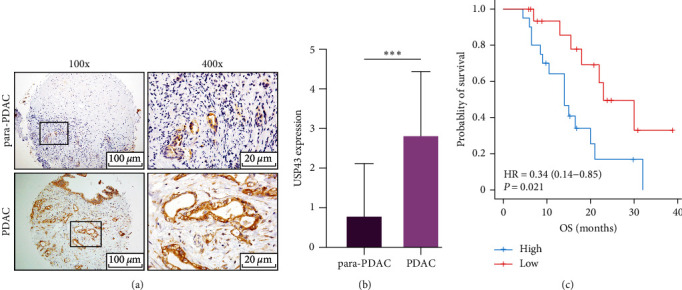
USP43 is highly expressed in PDAC and correlate with poor prognosis based on tissue microarray: (a) tissue microarray underwent IHC staining with USP43 antibody and we observe them with microscope (×100 and ×400); (b) after analyzed 38 patients' IHC staining, we analyzed the result with *t*-test; (c) survival curves of 38 patients with PDAC. Divide the patients according USP43 expression (high vs. low), then compare the OS of two group.  ^*∗∗∗*^*P*-values < 0.001.

To verify that the expression of USP43 significantly higher in tumor tissues, 38 PDAC tissues and 38 para-PDAC tissues were obtained from our hospital in this study. Interestingly, compared with normal tissue, the USP43 expression was significantly higher in tumor PDAC (*P* < 0.001, Figures 2(a) and 2(b)). After follow-up for 2 years, we divided the patients into two groups according to the median expression of USP43 (high vs. low), and we study the survival differences by using Kaplan–Meier curves that revealed the high USP43 expression patients were correlated with significantly shorter OS (*P* = 0.012, Figure 2(c)) confirming the bioinformatic result and indicating its potential role as a biomarker for PDAC patients. In addition, we investigated the association between USP43 expression and clinicopathological characteristics and serum biomarkers (Table 2). High USP43 expression was positively associated with surrounding organ infiltration (*P* = 0.044) and high CA19-9 level (*P* = 0.049) in tumor tissues, indicating that it may be a prognostic factor.

The univariate Cox proportional-hazards model revealed that three parameters, including USP43 expression (*P* = 0.019), CA19-9 (*P* = 0.005), and CA125 (*P* = 0.046), were substantially linked with increasing risk of death in patients with PDAC (Table 3). LMR (lymphocyte to monocyte ratio, *P* = 0.014) and lymphocyte in plasma (*P* = 0.039) were strongly linked with a reduced risk of mortality. Then, we build a multivariate Cox regression model for variables that were significant in the univariate analysis and show that only USP43 expression and CA19-9 were independent survival predictors (Table 4).

### 3.3. High USP43 Expression Promote Proliferation of PDAC

**Figure 3:**
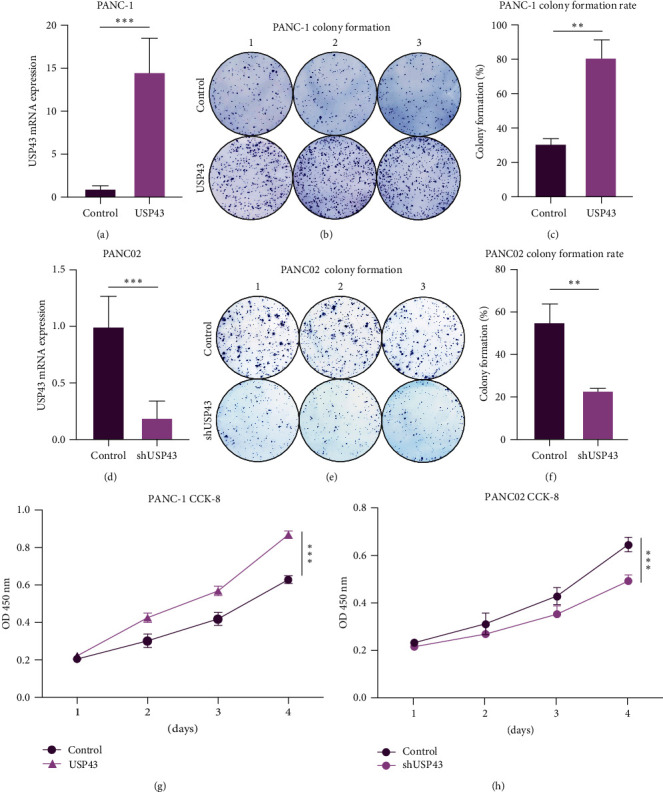
USP43 overexpression can promote proliferation of PDAC: (a) we contributed two cell lines, PANC-1 control group and USP43-OE group. We underwent qPCR to confirm efficiency of over expression; (b) cell colony formation with PANC-1 control group and USP43-OE group. We inoculated 500 cells into every well of six-well plate and culture for 5–10 days; (c) cell colony formation rate was calculated with the formula, (numbers of cell colony formation/500) × 100%; (d) using the same method, we contributed two cell lines, PANC02 control group and shUSP43 group. We underwent qPCR to confirm efficiency of knockdown; (e) cell colony formation with PANC02 control group and shUSP43 group. We inoculated 500 cells into every well of six-well plate and culture for 5–10 days; (f) cell colony formation rate was calculated with the formula, (numbers of cell colony formation/500) × 100%; (g) utilizing PANC-1 control group and USP43-OE group underwent CCK-8 arrays, and the absorbance was measured at OD 450 nm; (h) utilizing PANC02 control group and shUSP43 group underwent CCK-8 arrays, and the absorbance was measured at OD 450 nm.  ^*∗∗*^*P*-values < 0.01,  ^*∗∗∗*^*P*-values < 0.001.

At the cell lines level, comparing PANC-1 control group mRNA expression to PANC-1 USP43-OE group mRNA expression revealed significantly higher expression in the PANC-1 USP43-OE group (*P* < 0.001, Figure 3(a)). The USP43-OE group had a higher colony formation rate than the control group (*P* = 0.0018, Figure 3(b) and 3(c)) based on this result. In the interim, we conducted the colony formation assay with PANC02 control and PANC02 shUSP43 cells. It was demonstrated that the PANC02-shUSP43 group expressed significantly lower USP43 than the control group (*P* < 0.001, Figure 3(d)) among these two cell lines. Compared to the PANC02 control group, colony formation was significantly reduced in the PANC02 shUSP43 group (*P* = 0.0044, Figures 3(e) and 3(f)). In addition, CCK-8 assays were performed on PANC-1 (control-OE and USP43-OE) and PANC02 (control-sh and shUSP43) cell lines. The findings revealed that USP43-OE group dramatically enhanced the proliferation of PANC-1 cell lines (Figure 3(g)), and shUSP43 group remarkably decreased the proliferation of PANC02 cell lines (Figure 3(h)).

### 3.4. USP43-Related Genes and Biological Function

**Figure 4:**
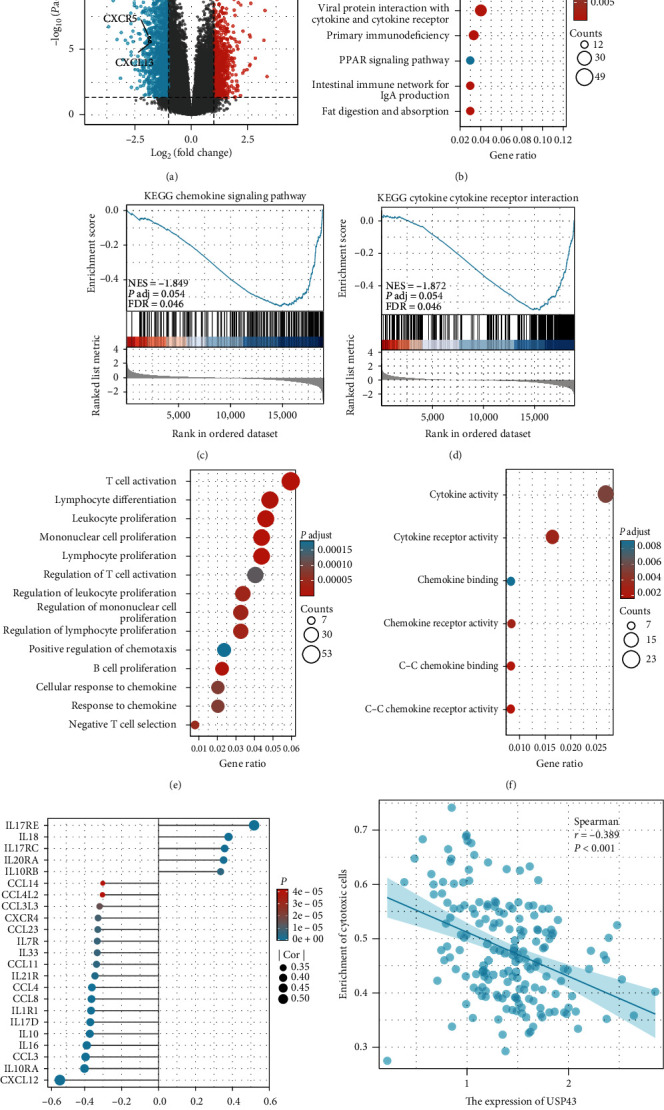
USP43 is associated with T cell activation and inhibits CD8^+^ T cell activation in PDAC based on TCGA database: (a) volcano plot of related genes differentially expressed in PDAC and normal pancreatic samples; (b) KEGG enrichment analysis was taken to find associated pathway or biology behavior; (c) KEGG enrichment analysis of the relationship between chemokine signaling pathway and USP43 expression; (d) KEGG enrichment analysis of the relationship between cytokine–cytokine receptor activity and USP43 expression; (e) GO enrichment analysis was taken and we observed that high USP43 expression related to many immunological biological processes; (f) the molecular function of USP43 associated with cytokine–cytokine receptor activity; (g) the correlation between USP43 and chemokines; (h) the correlation between USP43 and CTLs enrichment.

After confirming the predictive significance of USP43 using bioinformatics, patient samples, and cell lines, we attempted to investigate the underlying mechanism and linked genes that may explain its role. We discovered differentially expressed genes (DEGs) based on USP43 expression by examining the TCGA database, and numerous cytokine genes were negatively correlated with USP43 (Figure 4(a)). In addition to the DEGs, we performed a KEGG enrichment analysis and discovered that USP43 was strongly related with the chemokine signaling pathway and cytokine–cytokine receptor interaction (Figure 4(b)). Both associations are negative (*P* = 0.054, 0.054, respectively, Figures 4(c) and 4(d)). Furthermore, we conducted a GO enrichment analysis, which revealed that high USP43 expression is associated with a variety of immunological biological processes, including T cell activation, lymphocyte differentiation, and leukocyte proliferation (Figure 4(e)). Meanwhile, the molecular function of USP43 is linked to cytokine activity and cytokine receptor activity (Figure 4(f)). Because USP43 is so tightly associated with cytokines, we looked for which cytokines in the broad cytokine family were related to USP43 and found to be strongly inversely associated with CXCL12 expression (Figure 4(g)). Then, we looked at the association between USP43 and CTLs enrichment, and the results showed that they were inversely correlated (Figure 4(h)).

### 3.5. USP43 Inhibits CD8^+^ T Cell Activation in PDAC

**Figure 5:**
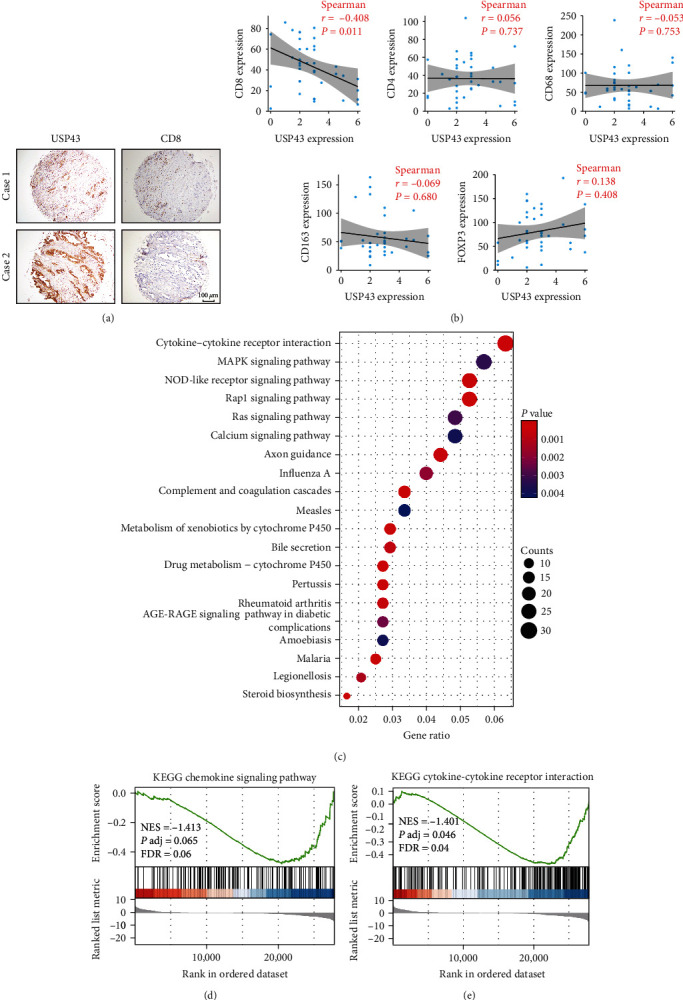
USP43 is associated with T cell activation and inhibits CD8^+^ T cell activation in PDAC based on tissue microarray: (a) IHC staining of 38 patients' tissue microarray showed the relationship between USP43 expression and CD8 expression; (b) correlation analysis of the expression of USP43 with CD4, CD8, CD68, CD163, and FOXP3 (Spearman correlation analysis); (c) utilizing PANC02 control and shUSP43 cell lines, we analyzed transcriptome of two cell lines to find the enrichment of DEGs; (d) the correlation between USP43 and chemokine signaling pathway; (e) the correlation between USP43 and cytokine–cytokine receptor interaction.

We examined the IHC stain of immune cell markers such as CD4, CD8, CD68, CD163, and FOXP3 in PDAC tissue to understand the immune-related function of the USP43 gene. The IHC results revealed that elevated USP43 expression was correlated negatively with CD8 expression (Figure 5(a)). The higher the expression of USP43, the lower the infiltration of CD8^+^ T cells. IHC staining of other markers revealed no significant association (Figure 5(b)). We used the PANC02 control and shUSP43 cell lines to show the association between USP43 and cytokines. We examined the transcriptomes of two cell lines and discovered that USP43 is indeed related to cytokine–cytokine receptor interaction (Figure 5(c)), with a negative tendency (Figures 5(d) and 5(e)). This finding supported the KEGG and GO enrichment analyses from the TCGA database, which indicated that the predictive value of USP43 gene may be owing to its role in immune modulation in PDAC patients.

## 4. Discussion

Patients with PDAC have a poor prognosis, highlighting the need to develop innovative therapeutic strategies for this aggressive illness [25]. USPs are the largest DUBs family and a growing number of studies show that various USPs may be important targets for cancer therapy due to their ability to regulate tumor formation, including tumor growth and the process of metastasis [16, 17, 19, 20, 26–28]. Previous research indicates that USP43, a member of the USPs family, plays a significant role in breast cancer, CRC, lung squamous cell carcinoma, and nonsmall cell lung cancer can promotes tumor migration, invasion, and associated with a poor prognosis [22, 29–31]. In osteosarcoma, the expression of USP43 is higher than normal tissue, but is not associated with prognosis [19].

In our study, we used the TCGA database and tissue microarrays of PDAC patients to assess the expression of USP43 in PDAC and para-PDAC/normal tissues. USP43 was shown to be overexpressed in PDAC tissues, and its high expression was related to decreased survival. High USP43 expression in tumor tissues was substantially associated with surrounding organ infiltration and elevated CA19-9 levels. Multiple Cox regression analysis also demonstrated that USP43 expression and CA19-9 level were independent predictive markers of PDAC, indicating that USP43 may be an essential adjunct in the diagnosis of PDAC. Experiments in vitro established that USP43 enhanced the proliferation of PDAC and that the overexpression or knockdown of USP43 promoted or inhibited the proliferation of PDAC, respectively. Therefore, elevated USP43 expression in PDAC patients suggested a poorer prognosis. USP43 malfunction may be one of the variables that influence the evolution of PDAC. Despite the complexity and diversity of USP43's underlying processes in PDAC patients, it remains an essential gene in carcinogenesis and development.

PDAC is characterized by a large number of stromal components and stromal cell infiltration which compose an immunosuppressive microenvironment. TIME of PDAC typically lacks of intratumor effector lymphocytes and results in a weak response to immunotherapy [8, 32, 33]. Therefore, PDAC is considered as a “cold tumor.” Previous investigations on infiltrating immune cells around PDAC determined that the ratio of CD8^+^ T cells was related to prolonged OS and RFS [34–37]. Poor tumor infiltration of CD8^+^ T cells results in low immunogenicity of PDAC, which is regarded as a significant factor in the failure of checkpoint immunotherapy in PDAC [38]. Thus, tumor-infiltrating CD8^+^ T cells may be able to predict the response of PDAC to ICIs treatment [39]. On the other hand, the CD3^+^, CD4^+^, and CD8^+^ T cells were independently related to tumor recurrence, according to previous literature [40, 41]. One study reported that, the high infiltration of CD3^+^, CD4^+^, and CD8^+^ T cells predicted a favorable OS [40]. It has been suggested that CD8^+^ T cells and NK cells can promote pyroptosis in cancer cells, and that pyroptosis of cancer cells might simultaneously trigger stronger anticancer immunity [42–44]. Similarly, it has been shown that cytotoxic T cells, such as CD8^+^ T cells and NK cells, might predict an increase in OS in samples with overexpression of pyroptosis and iron-induced targets [45]. In this study, we found that expression of USP43 was positively associated with poor prognosis and negatively associated with cytotoxic CD8^+^ T cell infiltration through IHC staining of tissue microarray and TCGA database. This also confirmed previous studies and further emphasized the anticancer effect of CD8^+^ T cell in PDAC.

Many studies have shown that CAFs possess the ability of promoting tumor proliferation and metastasis by secreting various cytokines [46, 47]. These studies suggest that targeting correlation between tumor and stromal components can improve treatment efficacy. Our study also confirmed that USP43 expression was negatively correlated with chemokine signaling pathway and with cytokine–cytokine receptor interactions.

In this research, we did not verify the relationship between USP43 expression and immune cells in vivo. In a more ideal situation, tumor cells should be implanted in mice. Second, the lack of clinical cases decreased the statistical power. Finally, the findings reported here need to be validated in further biological experiments.

In conclusion, our research indicates that the deubiquitinate function of USP43 may serve as a possible prognostic marker for PDAC. In addition, we discuss the possible role of USP43 in the development of TIME in PDAC. The transformation of a “cold tumor” into a “hot tumor” makes ICIs for PDAC conceivable [48, 49]. These discoveries not only complement to our current knowledge of TIME but also open the way for a potentially immunotherapy for PDAC.

## Figures and Tables

**Table 1:** Human and mouse primer sequences utilized in qPCR.

Characteristics	Forward primers (5′–3′)	Reverse primers (5′–3′)
*USP43* (human)	CAAGGCAATTCCCAGCACG	TGGTGACAGGCAGTTCTCAGA
*Usp43* (mouse)	AGCTCACGGGCTGGTATCT	AAGACCTGTACTGTGCTTGAAAG
*CXCL12* (human)	ATTCTCAACACTCCAAACTGTGC	ACTTTAGCTTCGGGTCAATGC
*Cxcl12* (mouse)	TGCATCAGTGACGGTAAACCA	TTCTTCAGCCGTGCAACAATC
*β-ACTIN* (human)	CATGTACGTTGCTATCCAGGC	CTCCTTAATGTCACGCACGAT
*β-Actin* (mouse)	GTGACGTTGACATCCGTAAAGA	GCCGGACTCATCGTACTCC

**Table 2:** *χ* ^2^ test to explore the relationship between USP43 expression and clinical features or tumor markers of PDAC.

Characteristics	Number	Tumoral USP43 expression		*P*-value
		Low	High	
Age (years old)				
≤64	20	12	8	0.194
>64	18	7	11	
Gender				
Male	19	8	11	0.330
Female	19	11	8	
T stage				
T1-2	23	12	11	0.740
T3-4	15	7	8	
N stage				
N0	20	9	11	0.516
N1-2	18	10	8	
Stage				
I–IIA	18	8	10	0.516
IIB–III	20	11	9	
Differentiation grade				
Well	9	2	7	0.160
Moderate	15	9	6	
Poor	14	8	6	
Surrounding organ infiltration				
Negative	14	10	4	0.044^*∗*^
Positive	24	9	15	
Surrounding fat infiltration				
Negative	19	10	9	0.746
Positive	19	9	10	
CA19-9 (U/ml)				
<307.5	22	14	8	0.049^*∗*^
>307.5	16	5	11	
CA125 (U/ml)				
<37.2	29	17	12	0.127
>37.2	9	2	7	
CEA (ng/ml)				
<4.66	27	16	11	0.074
>4.66	11	3	8	
AFP (IU/ml)				
<2.45	21	10	11	0.744
>2.45	17	9	8	

^*∗*^*P*-values < 0.05.

**Table 3:** Univariate Cox analyses of USP43 expression and clinicopathological parameters.

Characteristics	Univariate analysis		
	HR	95% CI	*P*-value
USP43 expression High expression vs. low	2.927	1.194–7.173	0.019^*∗*^
Surrounding organ infiltration No infiltration vs. infiltration	0.371	0.142–0.967	0.042^*∗*^
CA19-9 (U/ml) High vs. low	1.000	1.000–1.000	0.005^*∗*^
CA125 (U/ml) High vs. low	1.011	1.000–1.021	0.046^*∗*^
LMR High vs. low	0.748	0.592–0.944	0.014^*∗*^
Lymphocyte High vs. low	0.392	0.161–0.954	0.039^*∗*^

^*∗*^*P*-values < 0.05.

**Table 4:** Multivariate cox analyses of USP43 expression and clinicopathological parameters.

Characteristics	Multivariate analysis		
	HR	95%CI	*P*-value
USP43 expression High expression vs. low	2.652	1.055–6.663	0.038^*∗*^
Stage IIB–III vs. I–IIA	1.113	0.184–6.744	0.908
T stage T3–4 vs. T1–2	0.723	0.376–1.390	0.330
N stage N1–2 vs. N0	1.521	0.675–3.424	0.311
CA19-9 (U/ml) High vs. low	1.000	1.000–1.000	0.021^*∗*^
CA125 (U/ml) High vs. low	1.007	0.995–1.019	0.286
Lymphocyte High vs. low	0.503	0.197–1.285	0.151

^*∗*^*P*-values < 0.05.

## Data Availability

The online datasets for this study can be found in the Cancer Genome Atlas (TCGA-PAAD) (https://xenabrowser.net/datapages/, accessed December 16, 2022). The original contributions presented in the study are included in the article. Further inquiries can be directed to the corresponding authors.
